# Managing the Sugarcane Borer, *Diatraea saccharalis*, and Corn Earworm, *Helicoverpa zea*, using Bt Corn and Insecticide Treatments

**DOI:** 10.1673/031.013.10901

**Published:** 2013-10-22

**Authors:** Juliano R. Farias, Ervandil C. Costa, Jerson V. C. Guedes, Alessandro P. Arbage, Armando B. Neto, Mauricio Bigolin, Felipe F. Pinto

**Affiliations:** 1 Departamento de Defesa Fitossanitária, Universidade Federal de Santa Maria (UFSM), Av. Roraima, 1000, CEP: 97105-900, Santa Maria, RS, Brazil; 2 Departamento de Educação Agrícola e Extensão Rural, Universidade Federal de Santa Maria (UFSM), Av. Roraima, 1000, CEP: 97105-900, Santa Maria, RS, Brazil

**Keywords:** chemical control, Cry1Ab protein, genetically modified organism, integrated pest management

## Abstract

The sugarcane borer, *Diatraea saccharalis* (Fabricius) (Lepidoptera: Crambidae) and the corn earworm, *Helicoverpa zea* (Boddie) (Lepidoptera: Noctuidae), are important pests of corn in Brazil and have not been successfully managed, because of the difficulty of managing them with pesticides. The objective of this study was to evaluate the effect of Bt corn MON810, transformed with a gene from *Bacillus thuringiensis* Berliner (Bacillales: Bacillaceae) insecticide seed treatment, and foliar insecticide spray using treatments developed for control of the fall armyworm, *Spodoptera frugiperda* (J. E. Smith) (Lepidoptera: Noctuidae), which is the major pest of corn. The experiments were done under field conditions in early- and late-planted corn in the state of Rio Grande do Sul, Brazil, and in the laboratory. The MON810 corn reduced infestations and damage by *D. saccharalis* and *H. zea*. The insecticides used in seed treatments or foliar sprays did not affect *D. saccharalis* and *H. zea* infestations or damage levels. The exception was the insecticide seed treatment in non-transformed corn, which reduced early infestations of *D. saccharalis*. The MON810 corn, therefore, can be used for managing these two pest species, especially *D. saccharalis*.

## Introduction

The major insect pest of corn in Brazil has been the fall armyworm, *Spodoptera frugiperda* (J. E. Smith) (Lepidoptera: Noctuidae), which has been controlled mainly through insecticide treated seeds (Ceccon et al. 2004) or foliar insecticide sprays (Tomquelski and Martins 2007). Besides *S. frugiperda*, the sugarcane borer, *Diatraea saccharalis* (Fabricius) (Lepidoptera: Crambidae), and the corn earworm, *Helicoverpa zea* (Boddie) (Lepidoptera: Noctuidae), have caused significant damages to crops. However, due to the difficulty in controlling *D. saccharalis* and *H*. *zea* with insecticides, losses caused by them have generally been tolerated (Storer et al. 2001; Horner et al. 2003; Cruz 2007).

Over the past few years, Bt plants have been developed, receiving genes encoding insecticidal Cry proteins by molecular techniques from the *Bacillus thuringiensis* Berliner (Bacillales: Bacillaceae) bacterium (Bt). The first Bt corn hybrids commercialized in Brazil were the MON810 and Bt 11 events, both of which express the Cry1Ab protein (CTNBIO 2011). With the planting of Bt corn in Brazil, the damage caused by *D. saccharalis* and *H*. *zea* will likely decrease. Leaf damage was 5.3 times less by *D. saccharalis* in MON810 corn compared to its non-transformed isoline (Castro et al. 2004). The reduction in feeding on CrylAb corn ears by *H. zea* has been observed to range from 33% to 80% (Storer et al. 2001; Horner et al. 2003).

Brazil plants more than 12 million hectares of corn, 80% of which are hybrids of Bt corn. The planting of Bt corn in commercial areas of Brazil started in 2008. Almost nothing has been published on Bt corn for Brazilian weather conditions. The growing conditions for corn in Brazil are very diverse, ranging from subtropical areas in the south to tropical areas in the north and center of the country. Studies on the efficacy of Bt corn in Brazil can serve as a basis for the proper use of this technology with other forms of control, such as insecticides. The evaluation of *D. saccharalis* and *H. zea* can improve understanding of the development of resistance under Brazilian conditions. Such observations can help to plan the protection and longevity of Bt technology. Therefore, the objective of this study was to evaluate the effect of Bt corn MON810, insecticide in seed treatments, and foliar sprays, and their interactions, on the control of damage from *D*. *saccharalis* and *H. zea*.

## Materials and Methods

The field experiments were carried out with natural infestations of the insect pests at Itaara, Rio Grande do Sul state, Brazil, during the 2008/2009 crop season. In order to observe the isolated effects of both the seed treatment and MON810 corn in the survival of *D. saccharalis*, two experiments were carried out in laboratory conditions.

### Field experiments

The early-planted trial (September) was planted within a 50 ha area (29° 31′ 30,55′ S and 53° 44′ 17,98″ W) of commercial, nontransformed, no-till corn. The late-planted trial (December) was planted within an 81 ha area (29° 34′ 34,44″ S and 53° 46′ 25,51″ W) of commercial, no-till corn. The late-planted area contained approximately 60 ha of Bt corn, while the rest were non-transformed hybrids.

The experimental strip-split plot design was completely randomized, with four repetitions within a 2 × 2 × 2 factorial. The corn factor was represented by the non-transformed hybrids and the Bt MON810, the seed treatment by the presence or absence of insecticides, and foliar spray applications by the use or not of insecticides. The two levels of corn (nontransformed and Bt MON810), with the two levels of seed treatment (with and without seed treatment), were set out at random in the main plots, and the two levels of spray applications (with and without sprays) were set out at random in strips perpendicular to the main plots. The main plots measured 44 m × 8 m (16 seeded rows with a 0.5 m inter-row spacing), and the strips measured 32 m × 22 m.

The corn was planted on 18 September and 12 December 2008, for the early- and lateplanted trials respectively, with an inter-row spacing of 0.5 m and a density of 68,000 seeds ha^-1^. The corn hybrids used were (company - event): AS 1572 and AS 1572 Bt YG (Agroeste Sementes SA, www.agroeste.com.br/ - MON810 event) for early planting, and 3041 and 30A04 (Pioneer, www.pioneer.com - MON810 event) for late planting. The seed treatments were imidacloprid + thiodicarb (CropStar, Bayer, www.cropscience.bayer.com), at a rate of 45 + 135 g of a.i. on 60,000 seeds. The spray insecticides were novalurom + methomyl (Rimon 100 EC, Makhteshim Agan, www.ma-industries.com + Lannate BR, DuPont, www.dupont.com), at a rate of 15 + 129 g of a.i. ha^-1^ in early-planted corn, and methomyl (Lannate BR), at a rate of 129 g of a.i. ha^-1^, for the late-planted corn treatments.

The first foliar insecticide spray was made when more than 20% of the leaves of the nontransformed showed signs of *S. frugiperda* feeding. This occurred on 8 November 2008 for the early-planted corn, in the V7 growth stage (Ritchie et al. 1993), at 43 days after emergence. The first spray on the late-planted corn was made on 8 January 2009, in the V4 stage, at 21 days after emergence, with a second spray on 21 January 2009, at the V9 stage, at 34 days after emergence. The spraying equipment was self-propelled with a 24 m spray boom, equipped with 48 hydraulic fan nozzles, 110015 type, spaced at 0.5 m. All insecticide sprays were made with a spray volume of 200 L ha^-1^.

Evaluations were made on 10 plants randomly collected from each plot at the R4 stage (dough), corresponding to 111 and 100 days after emergence for the early- and late-planted treatments respectively. The number of *D*. *saccharalis* holes in the stems was counted, and the lengths of the galleries were measured. The percentage of stems attacked (infestation) was estimated from the presence of galleries greater than 0.30 cm in length. The number of holes, galleries, and gallery size (cm gallery^-1^) were obtained from the mean number of stems attacked. Ear damage was measured as the distance from the ear tip to the lesion extremities. The percentage of ears attacked (infestation) was calculated from the presence of the lesion (damage) and its size (cm ear^-1^), for an average of the number of ears attacked and considering the lesion from the tip of the ear.

### Laboratory experiments

The first laboratory experiment was to assess the seed treatment effect on *D. saccharalis*. The corn seed treatments were done with the insecticides imidacloprid + thiodicarb at a rate of 45 + 135 g de a.i. on 60,000 seeds. The corn hybrid DKB 350 (Dekalb, www.dekalb.com) was planted on plastic pots (10 L) containing a mixture of soil and vermiculite (1:1) and maintained in a greenhouse. The experiment was performed in acrylic plates (5.6 × 1 cm, diameter and height, respectively). Each tray received 7 ml of a 2.5% agar solution, a hard filter paper and pieces of corn leaf of each treatment. The treatmentswere with and without insecticide seed treatment tested at 10 and 20 days after plant emergence (DAE). For each treatment, 15 acrylic plates received 12 neonates each. Four days after the infestation, surviving larvae were evaluated.

The second experiment was performed to assess the Bt corn MON810 on *D. saccharalis*. The corn hybrids were DKB 350 and DKB 350 YG (Dekalb -**MON810** event). The corn planting and laboratory bioassay were similar to the previous lab experiment. The treatments were Bt and non-transformed corn tested in V_6_ stage (Ritchie et al. 1993). For each treatment, 10 acrylic plates received 12 neonates each. Four days after the infestation, surviving larvae were evaluated. These experiments were carried out in a climate chamber maintained at 27 ± 1° C, 60 ± 10% RH, and a 14:10 L:D photoperiod.

### Statistical analyses

The data from field experiments were submitted to an analysis of variance (F-test) at 5% probability considering three factors, namely corn (non-transformed and Bt MON810), seed treatment (with and without), and foliar sprays (with and without), verifying the isolated effect and the interactions of these factors. The data were analyzed as a strip-split plot design. The planting dates were not considered as a factor. The data from laboratory experiments were submitted to a *t-*test at 5% probability. The analyses were made using the R 2.14.1 program (R Development Core Team 2011).

## Results

There were no three-way interactions for corn, seed treatment, and foliar spray applications for early- and late-planted treatments for any of the variables analyzed for *D. saccharalis* and *H. zea* (Tables 1, 2, 4). A two-way interaction was observed just for corn and insecticide sprays for the response variable of holes caused by *D. saccharalis* in early-planted crops (Table 1).

The percentage of stems attacked by *D. saccharalis* was significantly lower for MON810 corn in both early-planted and late-planted trials, resulting in more than an 80% reduction in attacks for both planting periods (Table 1). Insecticide sprays for *S. frugiperda* did not affect the percentage of stems attacked by *D*. *saccharalis* in either the early- or late-planted corn treatments (Table 1).

**Table 1.**
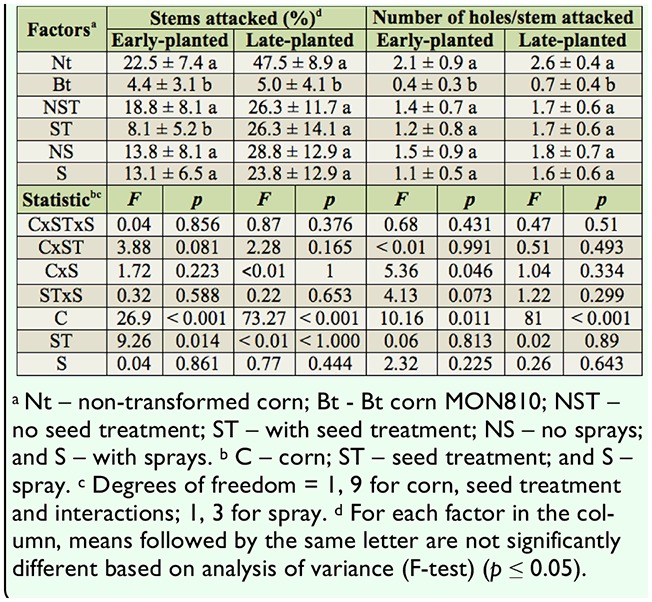
Stems attacked and number of holes per stem (mean ± standard error) caused by *Diatraea saccharalis* in corn. Itaara, Rio Grande do Sul state, Brazil, 2008/2009 crop season.

**Table 2.**
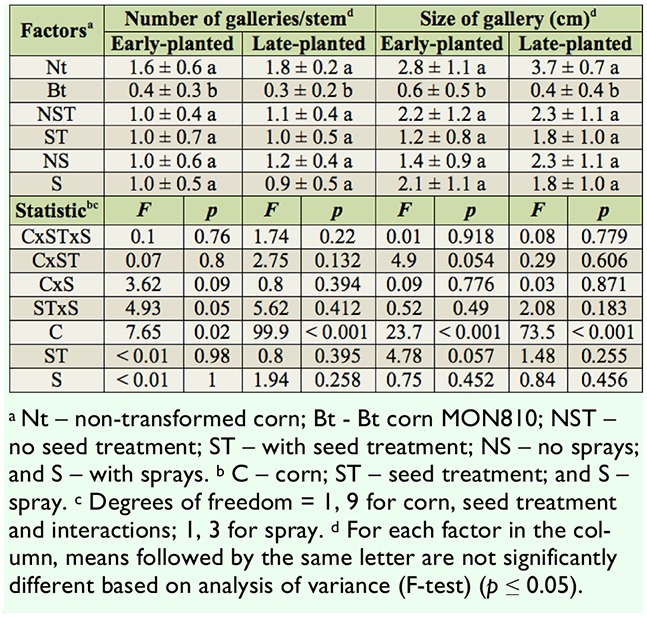
Number and size of galleries (mean ± standard error) in stems attacked by *Diatraea saccharalis* in corn. Itaara, Rio Grande do Sul state, Brazil, 2008/2009 cropseason.

Seed treatment resulted in a significant reduction in the number of stems attacked in the early-planted corn but not in the late-planted corn (Table 1). In early-planted corn, 13.8% of non-transformed corn stems were attacked by *D. saccharalis*, compared to 31.3% for the untreated seeds. The seed treatment effect in MON810 corn was less pronounced, with 2.5% and 6.3% of the stems of plants from seeds with and without treatment, respectively, attacked by *D. saccharalis* (data not shown in the tables).

The number of holes made by *D. saccharalis* was not significantly affected by seed treatment or by spraying. However, the expression of the Cry1Ab protein by the Bt corn caused a significant reduction in the number of orifices in the stems in both early- and late-planted corn (Table 1).

The number of galleries in stems attacked by *D. saccharalis* was significantly lower in the MON810 compared to the non-transformed corn in both early- and late-planted treatments . There were four to six times more galleries in the non-transformed corn for the early- and late-planted, respectively. Similarly, the size of the galleries made by *D. saccharalis* was larger in the non-transformed corn compared to the MON810 for both the early and late planting periods. Neither seed treatment nor insecticide sprays reduced the number and size of galleries made by *D. saccharalis* (Table 2).

In laboratory studies, Bt corn MON810 showed high efficacy against *D. saccharalis*. Two insects that survive at four days after inoculation were not able to cause a significant damage in leaf tissue of Bt corn MON810. The seed treatment caused significant mortality of *D. saccharalis* at 10 days after emergence . However, at 20 days after emergence the mortality of *D. saccharalis* was not significantly different in treated seed than in non-treated seed (Table3).

**Table 3.**
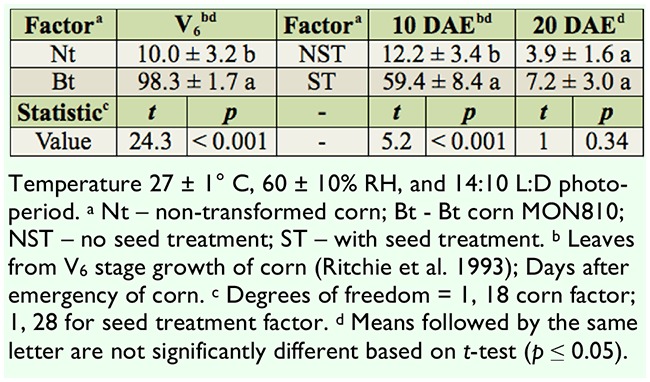
Mortality (mean ± standard error) of Diatraea saccharalisin corn.

**Table 4.**
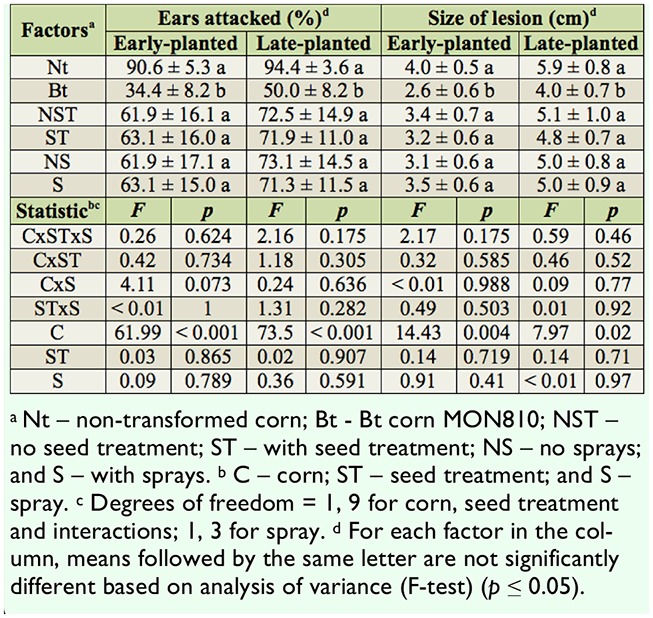
Ears attacked and size of lesion (mean ± standard error) in ears attacked by *Helicoverpa zea* in corn. Itaara, Rio Grande do Sul state, Brazil, 2008/2009 crop season.

The percentage of ears attacked by *H. zea* was significantly lower in the MON810 compared to the non-transformed corn in both early- and late-planted corn. The insecticides used in the seed treatment or sprays did not affect the percentage of ears attacked by *H. zea* in either planting period (Table 4), demonstrating that the lower attack by this species was due solely to the action of the Cry1Ab protein and not to the use of insecticides to control *S. frugiperda*.

The size of the lesion mfade by *H. zea* was not affected by the insectifcides used in the seed treatment of early- or late-planted corn or by sprays in early- or late-planted corn. However, there was a highly significant reduction in damage caused by the action of the CrylAb protein in early- and late-planted corn (Table4).

## Discussion

The only interaction observed between corn and insecticide spray can be explained by the number of factors used. Normally, increasing the number of factors can affect interactions for some variables. On the other hand, the absence of most interactions can be due to the moment that the insecticide treatments were applied. Another study observed interactions between hybrids (non-transformed and Bt corn MON810) and insecticides in two of five analyses for variables associated with *H. zea* (Farrar et al. 2009). The absence of an interaction in our study may be due to the crop stage when the insecticides were applied.

The expression of the CrylAb protein in MON810 corn was efficient in reducing *D*. *saccharalis* attack. It was observed that resistance was 5.3 to 5.4 times higher in MON810 corn compared to its nontransformed isoline for infestations in the V_6_ stage (Castro et al. 2004). Because attacks by *D. saccharalis* begin on the leaves, the expression of the CrylAb protein in the leaves of Bt plants was enough to reduce the percentage of insects that reached the stem. The results in the laboratory bioassay showed almost 100% mortality at four days after infestation. A similar result was observed by other authors under laboratory conditions at four days for *D. saccharalis* (Huang et al. 2006).

The stems of the MON810 corn showed fewer orifices made by *D. saccharalis*, but some were observed on the stems of these plants, confirmed by the presence of galleries. At least one previous study has shown that Btsusceptible *D. saccharalis* can cause small tunnels in the MON810 corn stems (Ghimire et al. 2011). This demonstrates that despite the high efficiency of this Bt event, some stem damage may occur and that the damage is not necessarily the result of Bt-resistant insects.

The gallery size of *D. saccharalis* in the MON810 corn was smaller, but despite the relatively low average size observed, there were galleries up to 4.4 cm long. The *D. saccharalis* larva rasps the corn leaf during the first two instars and afterwards perforates the stem and forms galleries within it (Cruz 2007). Therefore, when the larva reached the stem interior, it had already survived ingestion of the Cry1Ab protein expressed in the leaf, confirming that some larvae developed and could have completed their lifecycle in the MON810 corn. An alternate explanation may be that there were a small number of nonexpressing (non-transformed) corn plants within the MON810 plantings falling within commercial trait purity standards.

The infestation of *D. saccharalis* in earlyplanted corn was reduced by the insecticidal action of the seed treatment. The seed treatment insecticides used to control *S. frugiperda* also affected *D. saccharalis* (Cruz 2008). Among the seed treatment insecticides used to control *S. frugiperda*, one of the best is thiodicarb (Ceccon et al. 2004), which has resulted in more than 80% control for up to 14 days after emergence (Quintela et al. 2006). In the early-planted corn, the *D. saccharalis* infestation occurred earlier, and the larvae that hatched from this earlier infestation came into contact with the insecticide and died. Based on laboratory results, the infestation of *D*. *saccharalis* should be earlier than 20 days after emergence for seed treatment to have some efficacy.

Insecticide sprays to control *S. frugiperda* had no effect on the *D. saccharalis* infestation in either planting period. Similarly, Waquil et al. (1982) did not observe any influence in the percentage of stems attacked by *D. saccharalis* under insecticide treatments for *S*. *frugiperda*, including those using methomyl. The insecticides to control *S. frugiperda* can have an effect on *D. saccharalis*, but they need to come into contact with, or be ingested by, the larva when it is still on the outside of the stem. In our experiment, the insecticide sprays for *S. frugiperda* did not coincide with the presence of *D. saccharalis* on the outside of the stem, because the infestation occurred either before the sprays were applied or after the residual effects of the insecticide had worn off.

The percentage of ear damage caused by *H*. *zea* was lower in the MON810 corn, less than 50% of what it was in the non-transformed corn (90%). In the USA, studies have shown an almost 100% rate of ear damage in nontransformed corn by *H. zea*, compared to more than 63% damage in MON810 corn, with damage sometimes reaching 90% (Buntin 2008). This difference in the results for MON810 corn may be attributed to the genetic variability of the *H. zea* populations, resulting in differences in susceptibility. Another possible explanation is that *H. zea* popupopulations in the USA had already been in contact with the CrylAb protein for longer, whereas in our study this was the first year of large scale commercialization of the MON810 event in Brazil. This variability in percentage attack may also be related to the different hybrids used in these studies, because of possible Cry protein expression differences between hybrids.

The reductions of 35% and 32%, respectively, in the size of the lesions made by *H. zea* in the early- and late-plantings due to the action of the Cry1Ab protein in MON810 corn cannot be considered satisfactory. MON810 corn suppressed the establishment and development of the last instars of *H. zea* by at least 75%, but this level of control is unsatisfactory because it can increase the risk of evolution of resistance in areas where MON810 corn is widely planted (Horner et al. 2003). The insignificant reduction in lesion size made by *H*. *zea* can be explained by the results of Chilcutt et al. (2007), who found similar densities of 3rd and 4th instar larvae in both nontransformed and Bt corn, indicating that the 5th instar larvae, which cause less than half the damage, are affected by the CrylAb protein. Therefore, some larvae may have reached advanced stages and then died but had already caused lesions to the MON810 corn ears.

*H. zea* was unaffected by the insecticides used to control *S. frugiperda*. Similary, Waquil et al. (1982) did not observe differences in the percentage of ears attacked in insecticide-treated (including methomyl) and untreated control corn. Insecticide applications for *H*. *zea* control should be made immediately after adult oviposition so that the insecticide can reach the larvae while they are still on the outside of the ear and not protected by the leafy sheath. Therefore, insecticide sprays may affect *H. zea* as soon as the style-stigmas appear and not during the vegetative stage of the fcorn, when the insecticide sprays to control *S*. *frugiperda* were made in this experiment. Similarly, no effects of seed treatment on *H*. *zea* damage and infestation were expected, because the insecticidal effect in the seed is restricted to the first weeks after seedling emergence.

The results from this study can be considered from the point of view of the resistance of these species to the CrylAb protein and integrated pest management. Although these experiments are not specific for evaluating if the MON810 event is a high dose, the observed damage caused by both species suggests it may not fulfill the basic premise of the strategy of high dose/refuge to manage resistance. A previous study showed that CrylAb hybrids did not express a dose high enough to kill more than 95% of the resistant heterozygotes of *D. saccharalis* (Ghimire et al. 2011). Populations of *D. saccharalis* resistant to the CrylAb protein expressed in the corn plant also showed resistance to the Cry 1F protein (TC1507 event) (Ghimire et al. 2011). Thus, care must be taken to manage potential resistance to MON810 to prevent the loss of this event and other commercial Bt corn events. Part of this stewardship should include provisions for adequate refuge for the maintenance of susceptible populations.

From the point of view of integrated pest management, the MON810 corn reduced damage by *D. saccharalis* and *H. zea*. These species had been neglected before the commercial launch of these corn hybrids due to the difficulty in controlling them with insecticides. Therefore, the MON810 corn has incorporated a good management strategy into corn pest management. Seed treatment in the non-transformed corn resulted in an initial protection against *D. saccharalis* attack and, associated with other management strategies, may be used in integrated pest management due to the selectivity of this insecticide application method. However, the foliar sprays for *S. frugiperda* control are of little help in managing *D. saccharalis* and *H. zea*. Sprays for these two species should be made at very specific times, with a very narrow window of opportunity between the beginning of application and the moment when the larvae are protected. This makes correct application timing difficult and considerably reduces the efficiency of this strategy for these species.

This study is one of the first sources of information about the effect of Bt corn on pests that are not targets of insecticides, especially under Brazilian crop conditions. In addition, this information may allow for the planning and development of control measures that can improve the pest management system.
